# Quantification of high and low sEMG spectral components during sustained isometric contraction

**DOI:** 10.14814/phy2.15296

**Published:** 2022-05-25

**Authors:** Álvaro Costa‐García, Eduardo Iáñez, Moeka Yokoyama, Sayako Ueda, Shotaro Okajima, Shingo Shimoda

**Affiliations:** ^1^ CBS‐TOYOTA Collaboration Center in the Nagoya Science Park Research and Development Center Intelligent Behaviour Control Unit (RIKEN) Nagoya Aichi Japan; ^2^ Brain Machine Interface Systems Lab from Miguel Hernández University (UMH) Parque Cientifico UMH Edificio Innova Elche Alicante Spain

## Abstract

Superficial Electromyography (sEMG) spectrum contains aggregated information from several underlying physiological processes. Due to technological limitations, the isolation of these processes is challenging, and therefore, the interpretation of changes in muscle activity frequency is still controversial. Recent studies showed that the spectrum of sEMG signals recorded from isotonic and short‐term isometric contractions can be decomposed into independent components whose spectral features recall those of motor unit action potentials. In this paper sEMG spectral decomposition is tested during muscle fatigue induced by long‐term isometric contraction where sEMG spectral changes have been widely studied. The main goals of this work are to validate spectral component extraction during long‐term isometric muscle activation and the quantification of energy exchange between the low‐ and high‐frequency bands of sEMG signals during muscle fatigue.

## Graphical abstract

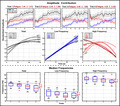

## 1 INTRODUCTION

Muscle activations can be quantified with wearable and increasingly cheap devices by the measurement of electromyography (EMG). Superficial EMG (sEMG) signals are the sum of motor unit action potentials (MUAPs) traveling along the sarcolemma of muscle fibers that trigger muscle contraction. While the analysis of temporal information of sEMG data is widely spread (De Luca, 1997), its evaluation in the frequency domain is less common due to different scientific discussions regarding the origins of sEMG spectrum. The history of this discussion is strongly connected with studies evaluating the role of slow and fast‐twitch muscle fibers during muscle fatigue.

Muscle fatigue is described as a decline in muscle performance after intensive muscle use (Enoka & Duchateau, 2008). Although its origins are still debated (Allen & Westerblad, 2004), in general, it is accepted that muscle fatigue is produced by the accumulation of lactic acid within skeletal muscle fibers, a subproduct of the anaerobic processes that generate energy in the form of ATP for muscle fiber contraction (Pedersen et al., 2003). Anaerobic processes are the main source of energy for fast‐twitch muscle fibers as they contain fewer mitochondrion and myoglobin proteins than slow‐twitch fibers whose main source of energy comes from cellular respiration (Allen et al., 2008). Several studies showed that fast fibers fatigued extremely quickly compared to slow fibers (Eberstein, 1963; Hill & Kupalov, 1929). Therefore, during sustained isometric contraction, the number of active fast fibers reduces at a faster rate than active slow fibers (Schiaffino & Reggiani, 2011).

In 1912, Piper et al. firstly reported a shift of sEMG spectrum toward lower frequencies during sustained isometric contraction (Piper, 1912). This phenomenon has been extensively reported in the literature as the temporal reduction of sEMG median frequency (Dimitrova & Dimitrov, 2003; Mannion & Dolan, 1994; Viitasalo & Komi, 1977). Although the analysis of the median frequency only provides an indirect measurement of the spectral shift, it has been proposed as an effective method for the detection of muscle fatigue (Cifrek et al., 2009; Coorevits et al., 2008).

It was first hypothesized that the change in the spectral properties was produced by changes in average muscle fiber conduction velocity (Arendt‐Nielsen et al., 1989; Merletti et al., 1990) related to the accumulation of lactic acid during continuous muscle contraction. Under this narrative, higher sEMG frequencies could be associated with a greater proportion of fast fibers (Pincivero et al., 2001; Rainoldi et al., 2008) and changes in the sEMG spectrum could be used to infer the recruitment of slower or faster motor units (Houtman et al., 2003; Bernardi et al., 1999).

This idea is still strongly debated. According to Farina, (2008), to establish a significant connection between sEMG spectrum and the recruitment of motor unit types, two assumptions need to be made. The first is that the conduction velocity of active motor units is related to fiber‐type proportions. Although there might be a certain level of connection, conduction velocity is also affected by motor unit discharge rates (Barry et al., 2007; Stalberg, 1966) and fiber size (Blijham et al., 2006; Kossev et al., 1992). Moreover, the distribution of muscle fiber type within motor unit pools has a very skewed distribution (both inter‐subject and inter‐muscle) (Elder et al., 1982). The second assumption is that spectral changes in sEMG signals are associated with variations in the average conduction velocity. This is also partially true, changes in the conduction velocity affect sEMG spectral features (Bigland‐Ritchie et al., 1981), however, this is not the only factor. Distance between fibers (Lindstrom & Magnusson, 1977), electrode location (Li & Sakamoto, 1996), the thickness of subcutaneous layers (Farina et al., 2002), fiber length and inclination (Dimitrov & Dimitrova, 1998), discharge rates of active motor units, and their degree of synchronization (Yao et al., 2000) have been also proven as factors affecting EMG spectral properties.

In 2004, Wakeling et al applied a principal component analysis to the spectrum of raw sEMG signals recorded over leg extensor muscles (Wakeling & Rozitis, 2004). According to their results, sEMG spectrum can be largely described by two principal components which behavior corresponded to the instances in which faster and slower motor units could be assumed active. In 2006, Tscharner et al. applied a similar technique to estimate the interplay between groups of fast and slow muscle fibers of the tibialis anterior and gastrocnemius muscle while running (Von Tscharner & Goepfert, 2006) with similar results regarding the extraction of two spectral components. In the last work, the authors state that “the terms fast and slow do not only refer to the conduction velocity but also to the shape of the motor unit action potential and are used to characterize the groups in a broader sense”. Therefore, independent spectral components were identified as groups or families of MUAPs whose shapes are significantly separated in the spectral domain. From this viewpoint, the spectral shift toward lower frequencies reported during muscle fatigue can be understood as the relative distribution of sEMG power between groups of spectrally separated MUAPs. The quantification of sEMG power associated with independent spectral components could provide a direct measurement of this spectral shift and shed more light on the processes behind muscle fatigue.

However, the spectral components extracted by Wakeling and Tscharner, came from sEMG data recorded from single electrodes during isotonic and short‐term isometric contractions. These muscle activations excluded two important factors influencing the shape of sEMG spectrum which are: (a) changes in the conduction velocity produced by longer‐term contractions and (b) signal propagation variabilities emerging from electrode location (thickness of subcutaneous layers, muscle fiber length, size, inclination, …). Therefore, before the characterization of muscle fatigue based on this spectral decomposition, it is necessary to evaluate the number of spectral components required to describe sEMG signals when these extra factors are included.

With the established background, current work pursues two goals. The first is to test the validity of the described spectral decomposition during muscle fatigue recorded from several locations of the same muscle. The second is the temporal characterization of the extracted spectral components during fatigue conditions. For this purpose, sEMG signals from the bicep muscle were recorded from 12 different locations using a medium‐density sEMG band during 30 s of high force isometric contractions. Spectral components were extracted using a Non‐Negative Matrix Factorization (NMF) from a time‐spectral analysis based on Fast‐Fourier Transform. The number of spectral components was selected to assure a reconstruction rate <80% computed from the Variance Accounted For (VAF). The separability of the spectral components was computed to further assess the validity of the decomposition. After the validation process, the temporal evolution of the spectral components during sustained muscle contraction was represented. From current literature, it is reasonable to expect that during sustained isometric contraction, there should be an exchange of signal energy from high to low frequencies. The application of the proposed spectral analysis should provide a direct way to measure this exchange, not as the relative spectral shifting provided by the evaluation of the median frequency, but by the actual quantification of the amount of spectral energy associated with high‐ and low‐frequency bands.

## 2 MATERIALS AND METHODS

### 2.1 EMG band

**FIGURE 1:**
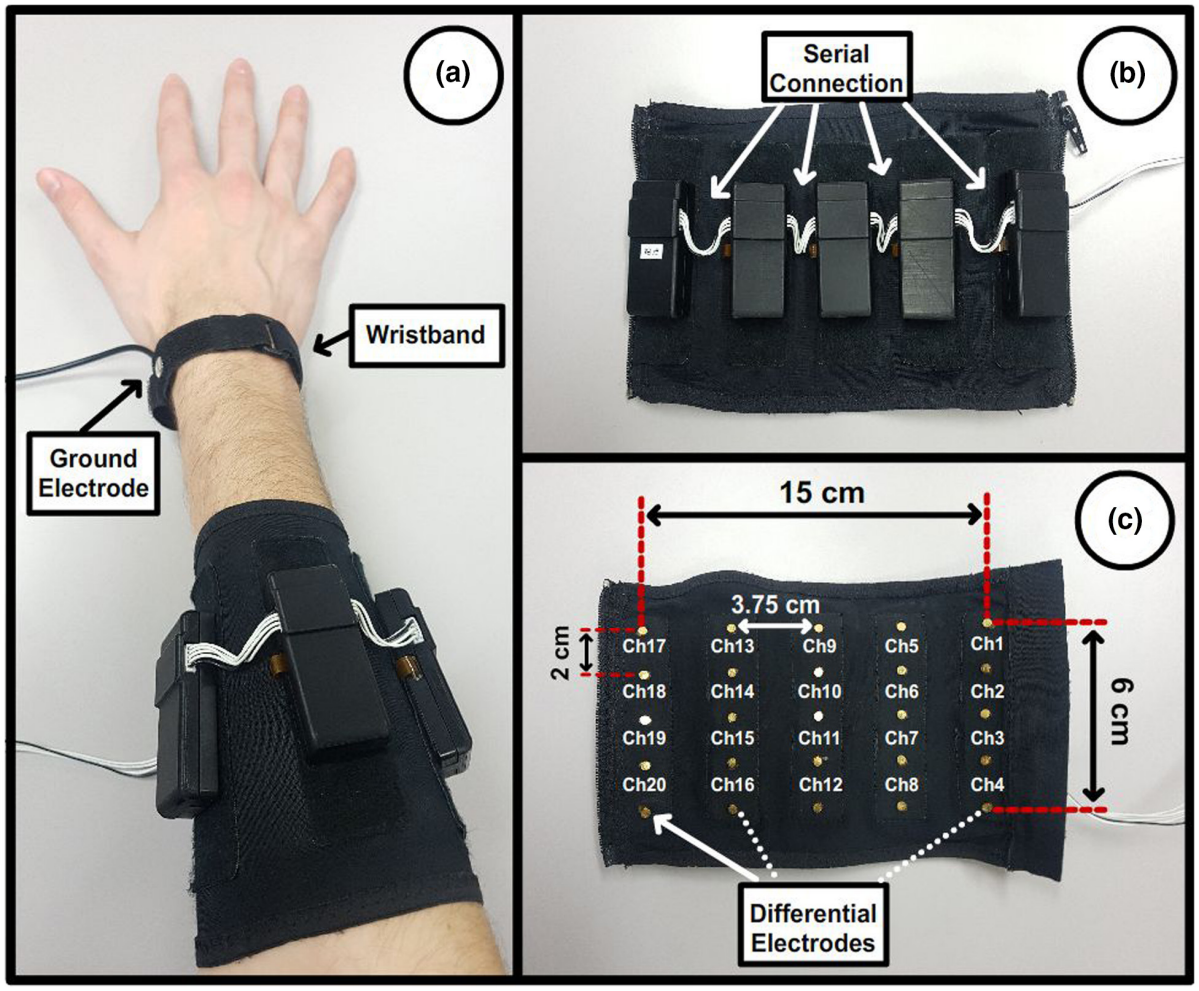
EMG band. Composed of a 5 × 5 matrix of differential electrodes that simultaneously records a matrix of 5 × 4 channels. All electrodes are referenced to a ground located on the wrist through a wristband.

An elastic medium‐density electrode band was used to record sEMG signals around the forearm. The band contained 25 dry electrodes distributed in five arrays and grounded via a wristband. EMG signals were obtained as the differential signal between each pair of vertically consecutive electrodes, which provides a 4 × 5 matrix of spatially distributed signals. Signals were digitized at 2000 Hz and sent to a PC through a USB connector (Figure 1). The electronics were powered through a small battery and did not include hardware filters.

### 2.2 Experimental protocol

**FIGURE 2:**
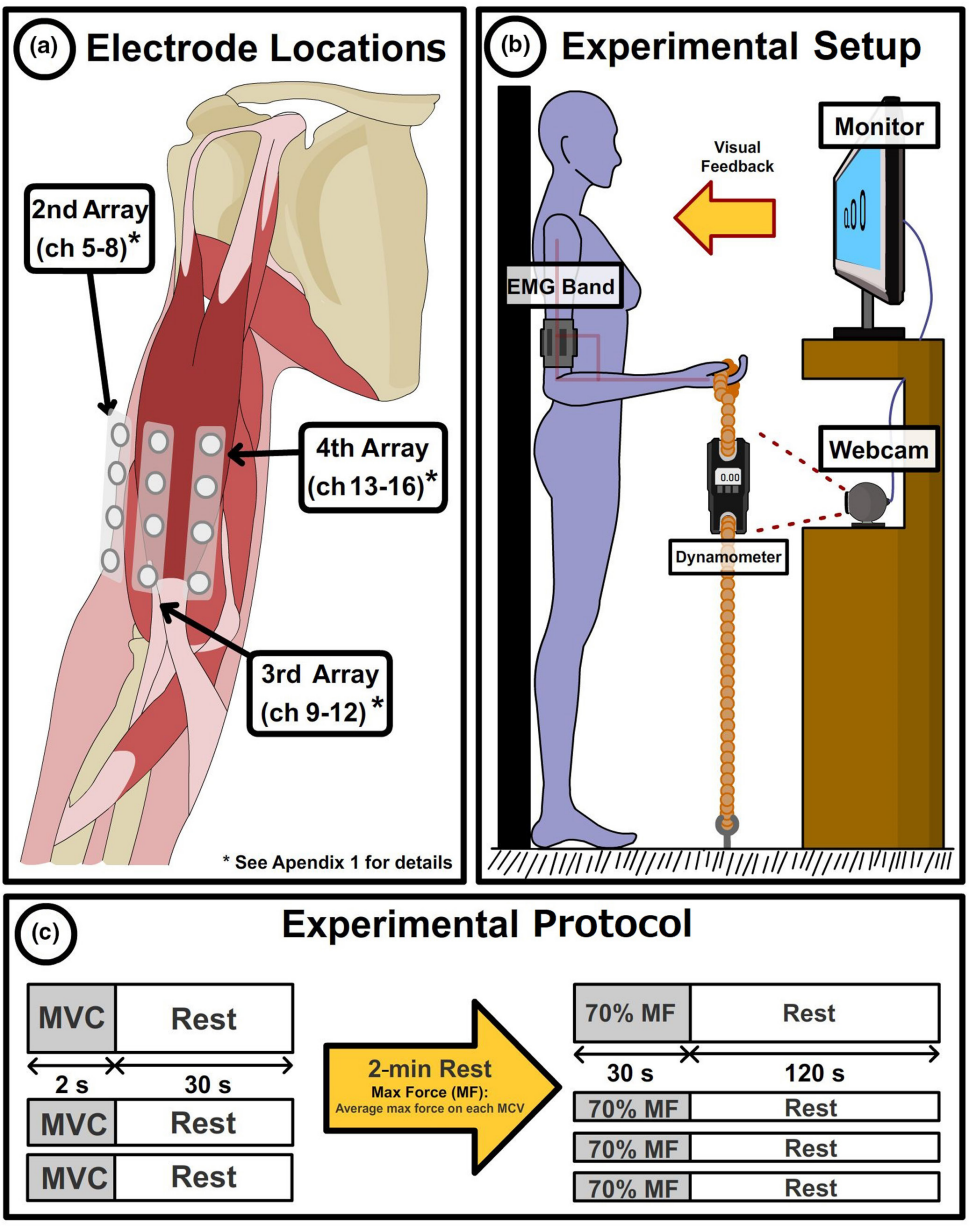
Experimental setup and protocol. (a) Graphical representation of sEMG acquisition points from the biceps muscle. (b) Experimental environment and setup. A subject stood with their back against a wall and performed isometric biceps contraction by pulling a rope attached to a dynamometer. The subject received feedback on the force exerted via a visual interface that obtained the data from a webcam monitoring the dynamometer.

The surface EMG band was placed over a subject’s biceps brachii muscle (see Appendix 1 for detailed information on the placement protocol). Channels 5–16 were used to record sEMG data, spatially shifted as shown in Figure 2(a). The subject stood against a wall while holding a dynamometer, which measures the generated force in kilograms (resolution 0.1 kg). The dynamometer was fixed to the floor via a rope, whose length was adjusted to keep the subject’s elbow flexed at 90 degrees. Real‐time information on the exerted force was provided to the subject through a screen that projected the visual information obtained from the dynamometer using a webcam (Figure 2b). The experimental protocol is presented in Figure 2(c). During the first part of the experiment, subjects were asked to perform three isometric contractions with maximum effort for 2 s with 10 s of rest between contractions. The maximum force exerted by each subject was computed as the average of the maximum force values recorded for each contraction. In the second part of the experiment, subjects were asked to perform an isometric contraction for 30 s maintaining 70% of their maximum force. This process was repeated four times with 1 min of rest between trials. Before each trial was started, subjective fatigue was measured using the 11‐point scale proposed by Kim et al. (2010), where 0 represents no fatigue at all and 10 represents the worst possible fatigue.

### 2.3 Participants

Nine healthy individuals participated in the experiment: Four women and five men aged 27–45 years (34.88 ± 6.71).

### 2.4 Methodology workflow

**FIGURE 3:**
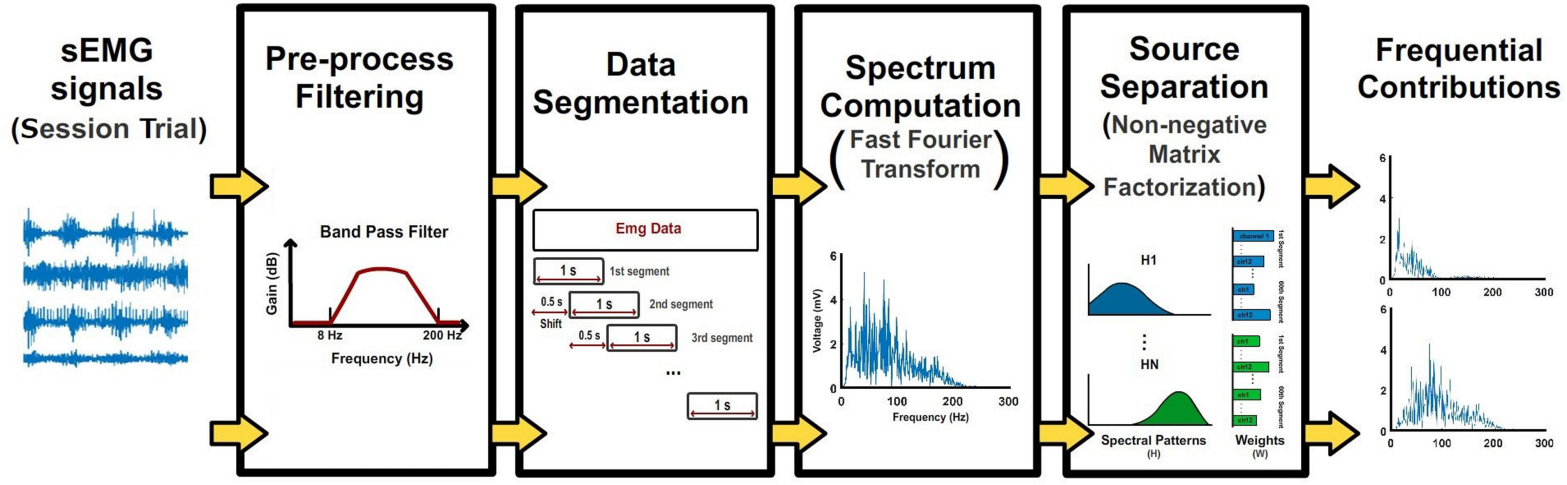
Overall sEMG signal processing. Raw sEMG signals were notch‐filtered at 60 Hz and bandpass‐filtered between 8 and 200 Hz. Filtered data were segmented in 1‐s epochs with 0.5 s of overlap. The spectrum of each channel and epoch was computed. The non‐negative matrix factorization algorithm was used to extract common sources from the set of spectra.

Figure 3 summarizes the methodology used to extract the spectral components from the sEMG signals recorded during an experimental trial. All channels were bandpass filter between 8 and 200 Hz. After filtering, the data were divided into 1‐s segments with 0.5 s of overlap between segments, and the spectrum of each segment was computed using a Fast Fourier Transform (FFT). Finally, spectral components were extracted using the NMF algorithm. Each stage is described in detail in the following sections.

### 2.5 Data segmentation

**FIGURE 4:**
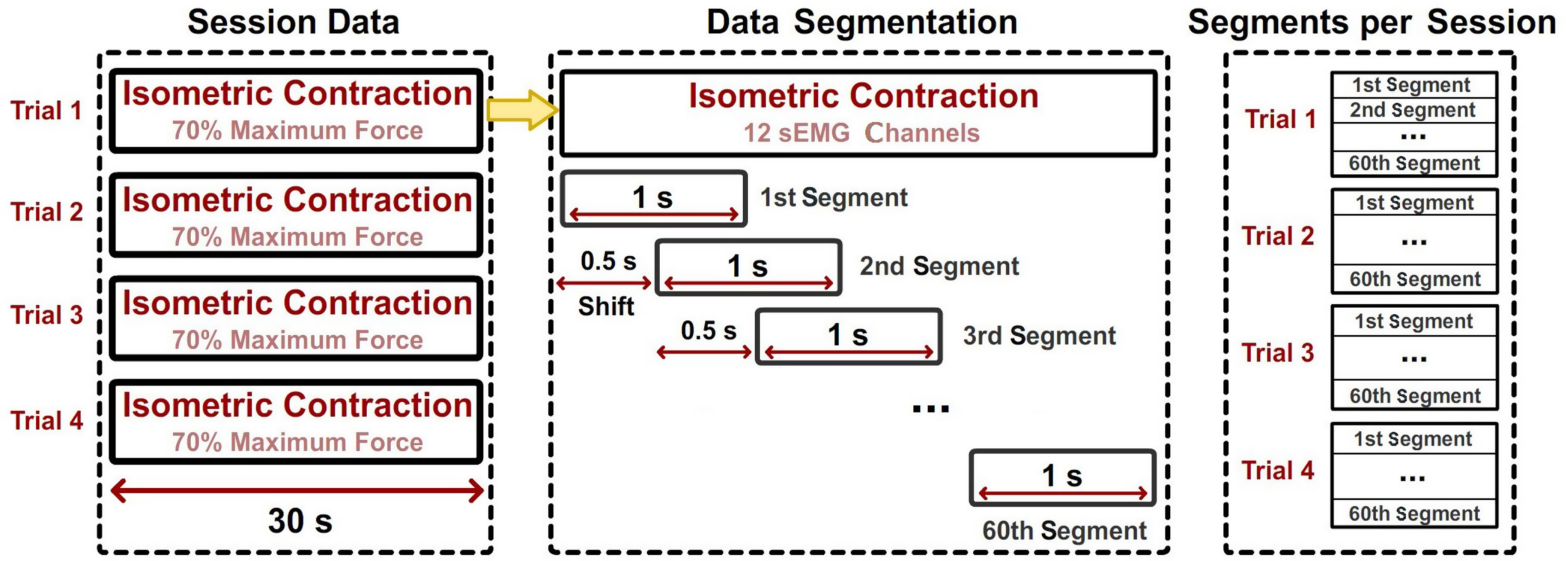
Data segmentation. Protocol used to segment the data. Each trial containing 30 s of isometric biceps contraction at 70% of the maximum force was divided into 1‐s epochs with an overlap of 0.5 s. In total, 60 epochs were extracted from a single trial.

For each experimental session, the recorded sEMG was divided into four trials of 30 s of isometric contraction in which the subjects were generating 70% of their maximum force (Figure 4—Session Data). Each trial contained information on the muscle activity produced by the biceps muscle recorded from 12 spatially shifted channels (see Appendix 1 for details). To obtain temporally shifted information, each trial was divided into 60 segments of 1 s each (2000 samples) with a shift of 0.5 s between segments, as illustrated in Figure 4 (data segmentation). The segment length was selected to ensure that the frequency range of interest will not be affected by signal aliasing.

### 2.6 sEMG preprocessing

Prior to signal segmentation, a Butterworth 1‐Hz high‐pass filter was applied in each trial to remove the DC component of the signal. For signals recorded at a sampling frequency of 2000 Hz, frequencies lower than 1000 Hz are considered free from aliasing effects. However, if the sampling frequency is much higher than the natural frequencies of the signal under analysis, the evaluation of very high frequencies might be unnecessary. Instead of using the typical frequency ranges accepted for sEMG data by the literature, in the present work, the spectral properties of the recorded data set were pre‐analyzed to select the spectral band of interest.

**FIGURE 5:**
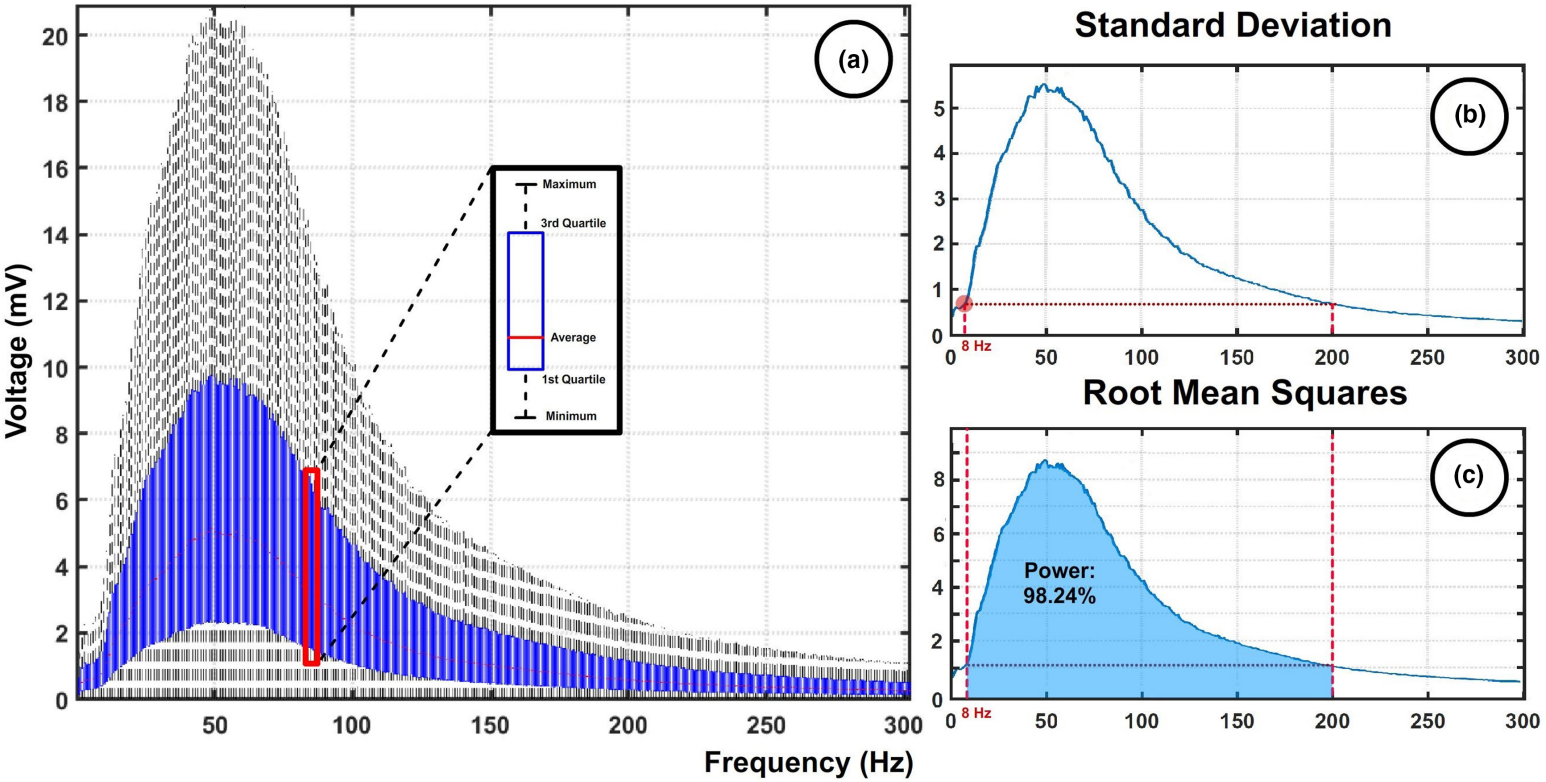
Data set spectral evaluation. (a) Graph containing the spectral information extracted from 18,570 signals (9 subjects × 4 trials × 60 epochs ×12 channels). (b) The standard deviation is computed for each frequency. (c) The energy per unit of time contained in each frequency is computed as the root mean square.

From each experimental session (4 trials), a total of 240 temporally shifted sEMG segments were extracted. Each of these segments contained 12 channels with spatially shifted information, giving 240 × 5 = 2080 sEMG epochs per session. Moreover, the experiment was undertaken by nine subjects, giving a total of 18,570 sEMG epochs. The spectrum of each of these epochs was computed by fast Fourier transform (FFT) and the set of values obtained for each frequency is presented in Figure 5(a) in the form of a boxplot.

To determine which frequency range contained relevant information for analysis, two factors were evaluated. The first was the standard deviation of the data for each frequency. The biological processes under evaluation generate widely reported frequency shifts that will cause higher variations in relevant frequencies (Figure 5b). The second parameter was the power generated. To ensure that the data under analysis properly represent the generated muscle activity, the range of frequencies selected should contain a significant percentage of the total power of the sEMG signal (Figure 5c). Both power and standard deviation values showed relevant growth after 8 Hz. The values of these parameters at 8 Hz were used as a threshold to select the range of frequencies, as shown in Figure 5(c). Based on this evaluation, sEMG data were bandpass filtered between 8 and 200 Hz using a Butterworth filter. The power contained in this frequency band represented 98.24% of the total power of the signals evaluated.

### 2.7 Source separation

**FIGURE 6:**
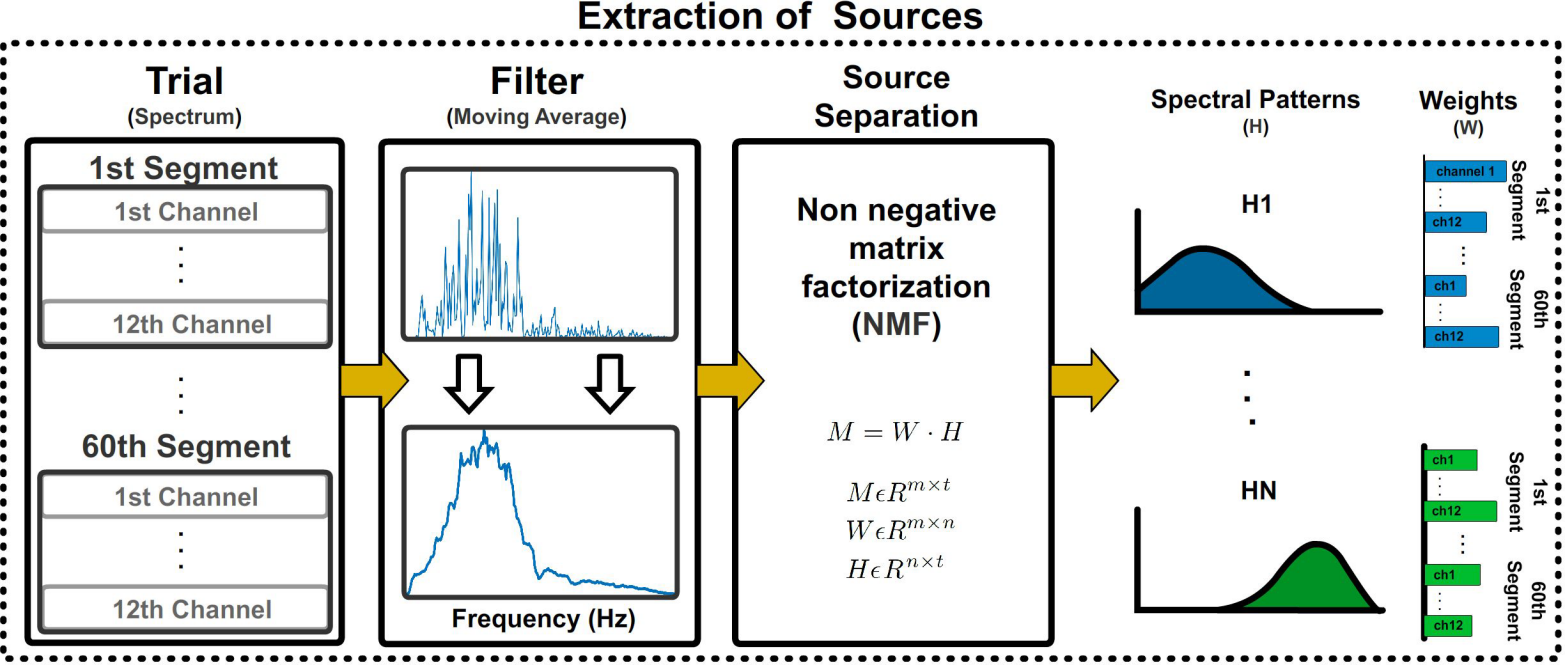
Source separation algorithm. The spectrum of each epoch and channel was computed by using a Fast Fourier transform and a moving average filter was applied to highlight its envelope. The non‐negative matrix factorization algorithm was used to describe all epochs (from all channels) contained in a single trial with *n* spectral components.

**FIGURE 7:**
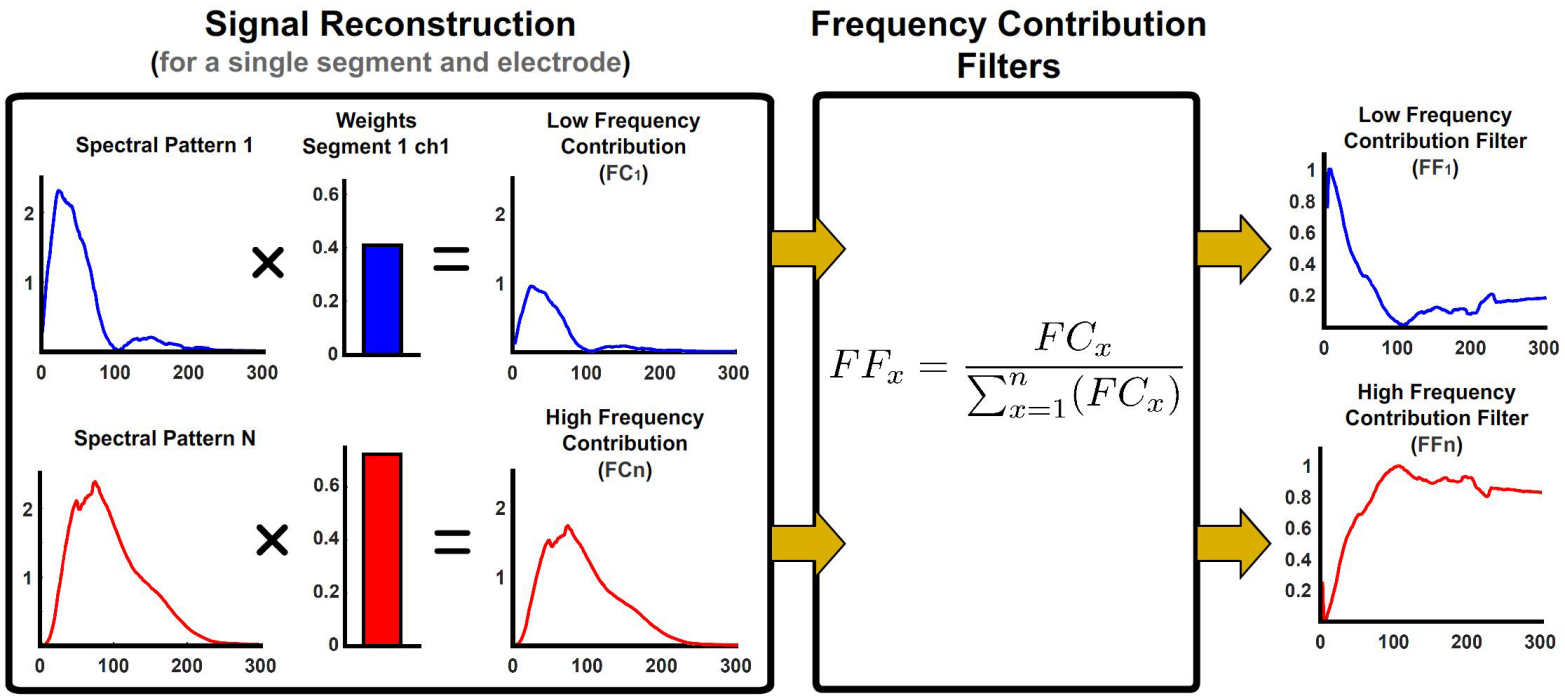
Filters for spectrum reconstruction. The envelope contribution of each component to each epoch was used to define filters allowing the extraction of each component from the raw signal spectrum.

**FIGURE 8:**
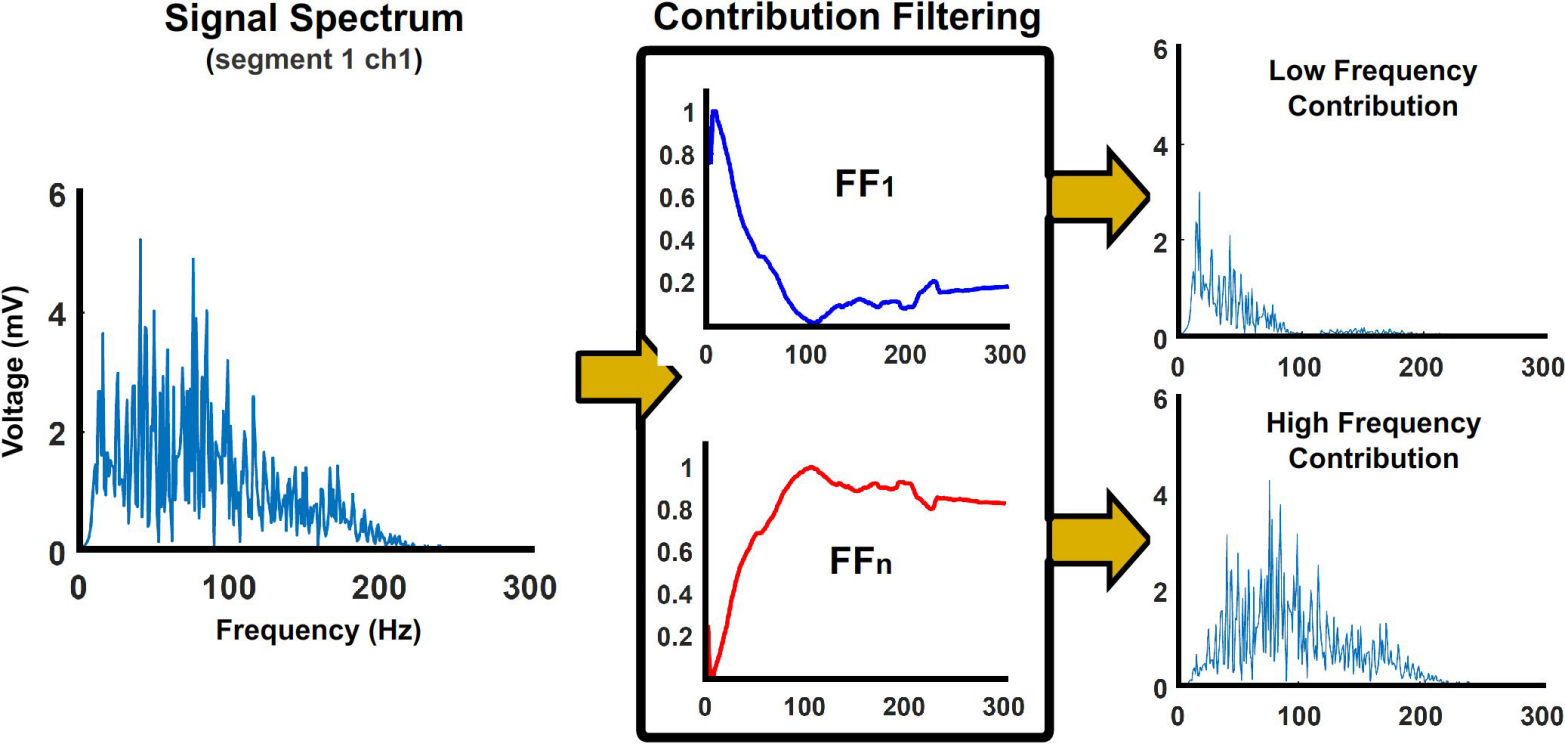
Extraction of spectral contributions. Filters computed in Figure 7 were applied to individual spectra to extract the non‐enveloped contribution associated with each spectral component.

Figure 6 shows the steps followed to decompose a set of signal spectrums. This process was applied over all segments of one trial (60 segments of 1 s containing information from 12 channels). Prior to source separation, a moving average filter was applied to the spectrum to emphasize its envelope. Subsequently, the spectrum of sEMG signals was described as:

(1) M=W·H,

(2) $$M\epsilon R^{m\times f},W\epsilon R^{m\times n},H\epsilon R^{n\times f},$$

where *M* is an *m* × *f* matrix of sEMG spectral data (with *m* the number of epochs and *f* the frequencies under analysis), *W* is an *m* × *n* matrix containing the weights associated with each spectral component used to reduce the *m* segments to an *n*‐dimensional space, and *H* is an *n* × *f* matrix containing the shape of the spectral components. Matrices *W* and *H* can be calculated from M through the Non‐negative Matrix Factorization (NMF) algorithm by fixing the n‐dimensionality. The NMF algorithm extracts H components by minimizing the correlation between them (Zhang & Fang, 2007). Using the matrices W and H, it is possible to obtain the envelope contribution from the extracted spectral components to each segment evaluated, as shown in Figure 7. These envelopes were used to compute frequency contributions filters ($FF_{x}$) as follows:

(3) $$FF_{x}=\frac{\left(FC_{x}\right)}{\sum_{x=1}^{n}\left(FC_{x}\right)},$$

where *FC_x_* is the envelope contribution of the spectral component *x* and *n* is the total number of spectral components. Finally, Figure 8 shows how the computed filters were used to extract the different frequency contributions from the original spectrum of each segment and channel.

### 2.8 Spectral component assessment

The number *n* of spectral components was selected as the lowest value that allows the recovery of the original data with a reconstruction rate >80% according to the Variance Accounted For (VAF). The separability between spectral components was quantified by their Bhattacharyya distance (Bdist) firstly introduced by Bhattacharyya, (1946). This parameter is closely related to the Bhattacharyya coefficient which measures the amount of overlap between two statistical samples or populations. Since the Bdist can provide any value in the range 0 <* Bdist *< ∞, a selection criterion is necessary. In Choi and Lee (2003), Choi and Lee studied the Bayes error (Antos et al., 1999) of 2 tasks classification against the Bhattacharyya distance between both classes. As a result, they obtain a logarithmic behavior being the Bayes error between ~0% and 2% for *Bdist *> 3.5. This value will be used as a threshold to define each pair of groups of the spectral component as highly separable. In addition, the significance between spectral components’ median frequencies was assessed by a Wilcoxon sum‐rank test with a confidence interval of 95 (Steel, 1959). A different Wilcoxon sum‐rank test was run over the spectral component median frequencies grouped both by subjects (to show inter‐subject variabilities) and trials (to show fatigue‐related variabilities). A Bonferroni‐Holms correction for multiple comparisons (Abdi, 2010) was applied to remove multiple comparison‐related biases.

## 3 RESULTS

### 3.1 Spectral components

**FIGURE 9:**
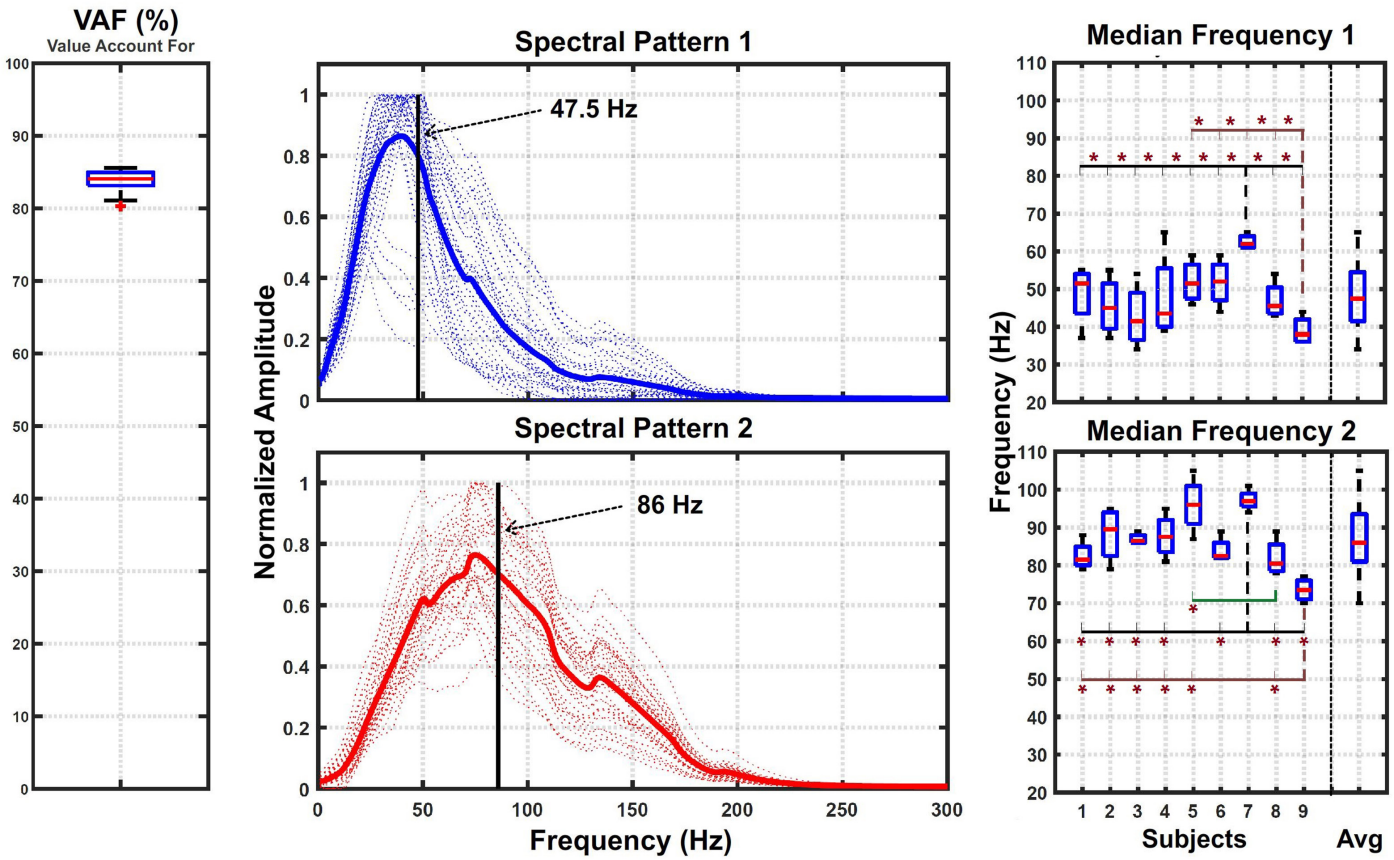
Spectral component features. The graph on the left shows a boxplot representation of the VAF values obtained from all trials. Middle graphs show the shape of the spectral components extracted from single trials and their average value. Components were normalized by a single maximum computed from each pair. Graphs on the right show boxplot representations of the components’ median frequencies for the whole set of data and for each subject.

Two spectral components were extracted from the set of signals recorded during each trial. After data segmentation, the total number of signals evaluated in each trial was 60 *segments* × 12 *channels* =720 signals of 1‐s duration each. The features of the spectral components extracted are represented in Figure 9. The first graph on the left shows the Value Account For (VAF) representing the robustness of using two components to reconstruct the original signals. The graph shows that the signals extracted from each trial (60 *segments* × 12 *channels* =720 signals of 1‐s duration each) can be reconstructed with a VAF of 83.84 ± 1.36%. The two middle graphs show the average of the two normalized spectral components together with their median frequencies. In addition, the median frequencies recorded from each subject are represented in the two graphs on the right. Inter‐subject statistical significance represented by an asterisk shows relevant differences for subjects 7 and 9. The last boxplot on the right represents the total distributions of the median frequencies, with a value of 47.55 ± 8.91 Hz for the low‐frequency component and 86.47 ± 8.15 Hz for the high‐frequency component. The separability analysis provided a Bdist = 21.16 between both sets of spectral components which largely exceed the threshold criteria (Bdist > 3.5) for highly separable classes. Moreover, the statistical Wilcoxon sum‐rank test showed significant differences between the median frequencies recorded from each spectral component group.

### 3.2 Components’ contribution to the signal energy

**FIGURE 10:**
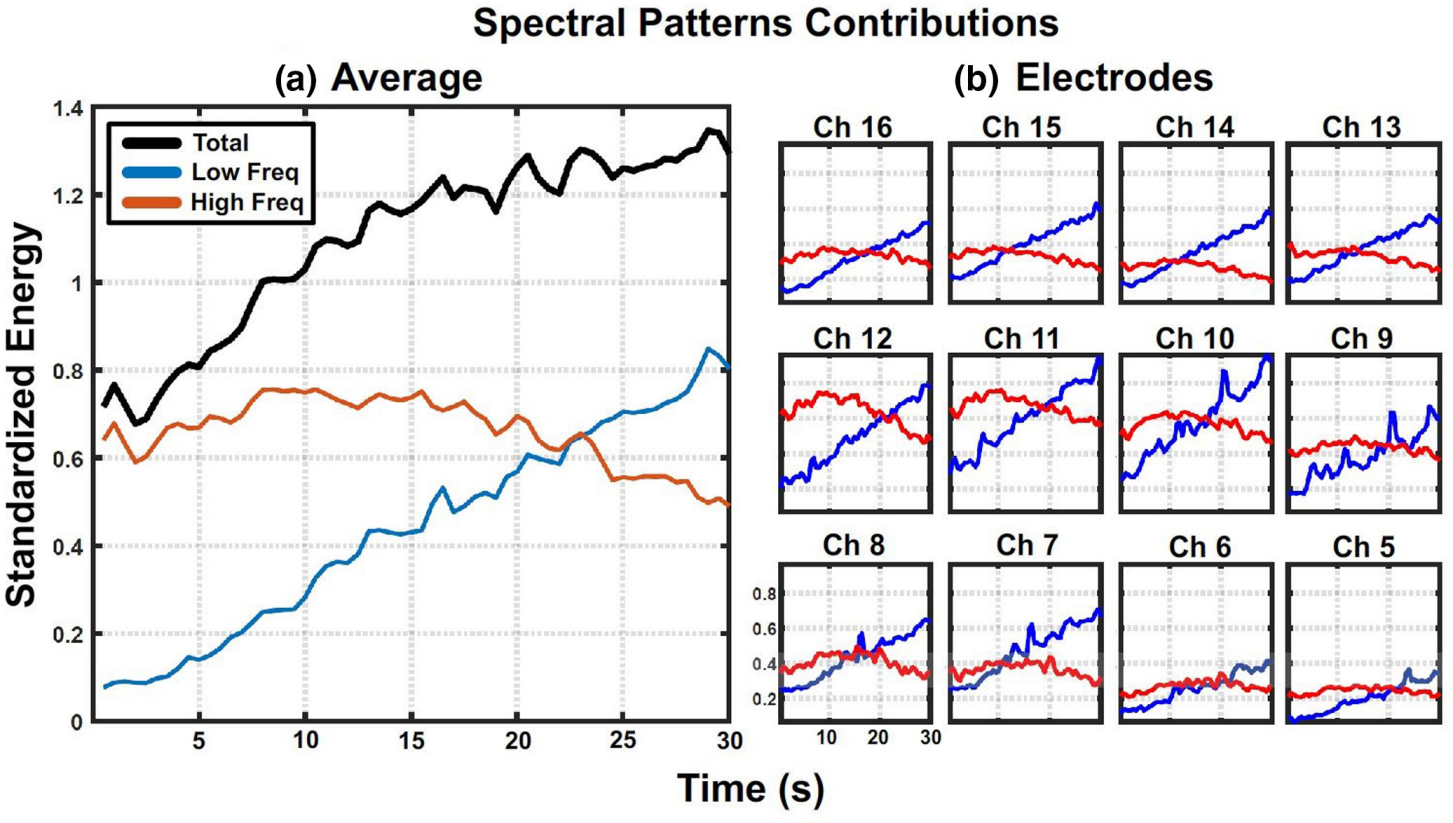
Signal’s energy distribution during contraction. Evolution of sEMG energy carried by each spectral component during the 30 s of isometric contraction. The graph on the left shows the average of all channels, subjects, and trials while the graphs on the right show the average of all subjects and trials for each channel.

After the spectral decomposition shown in Figure 8(a), the energy of the low‐ and high‐frequency components was computed as the area under the curve for each segment. These values are aligned in time and represented in Figure 10 to show the temporal evolution in both frequency components during the maintained isometric contraction. The black line in the left graph represents the evolution of the energy of the sEMG data during the 30 s in which subjects were maintaining 70% of the maximum generated force through isometric contraction of the biceps. In addition, the graph shows how low‐frequency (red line) and high‐frequency (blue line) spectral components contributed to the total energy during the motion. This graph was computed as the average of all subjects, trials, and electrodes. Results show a progressive increase in the signal’s total energy during sustained isometric contraction which is also a widely reported phenomenon (Cobb & Forbes, 1923). At the beginning of the contraction, most of the signal’s energy comes from the high‐frequency component, and both components present energy increase during 5–10 s. After that point, low‐frequency energy keeps increasing linearly while high‐frequency components start decreasing. After 20–25 s of contraction low‐frequency, energy contribution surpasses high‐frequency contribution. Figure 8(b) shows the energy distribution for each electrode across subjects and trials. Although each channel shows slightly different amplitude and component’s crossing points, all of them show the same trend described in Figure 8(a).

### 3.3 Components’ energy distribution with fatigue

**FIGURE 11:**
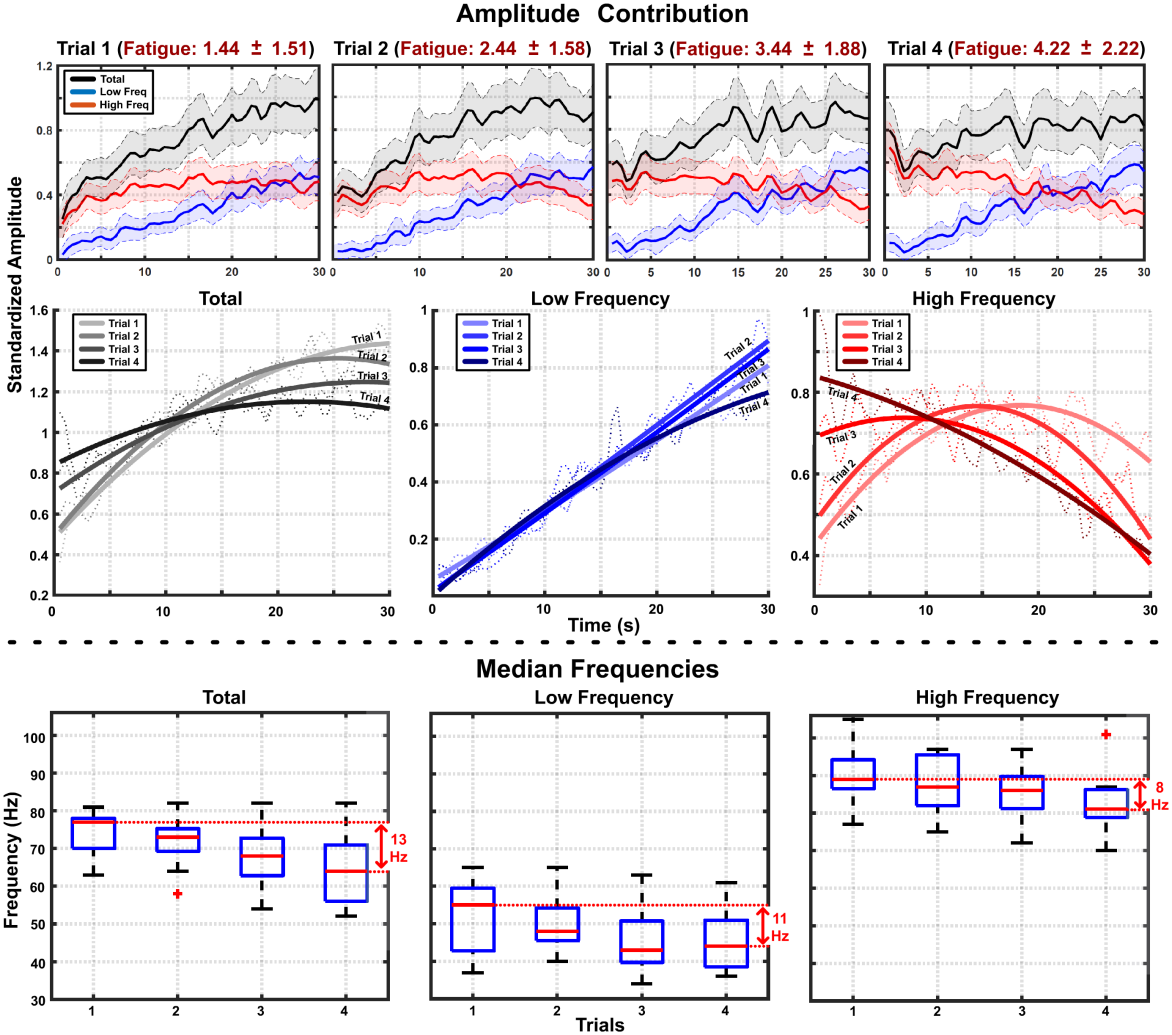
Signal’s energy distribution depending on initial fatigue. The four graphs at the top show the temporal evolution in the energy of both spectral components averaged by trial together with their respective standard deviation. In each graph, the title shows the average and standard deviation of the subjective fatigue reported by all subjects at the beginning of the trial. The graphs in the center row separately show the total energy (left), the energy of the low‐frequency component (middle), and the energy of the high‐frequency component (right) for each trial. The graphs at the bottom show the median frequencies of the total signal spectrum and the median frequencies of each spectral component arranged by trial.

Figure 11 summarizes the effects of initial fatigue level on the energy distribution between both spectral components. The upper graphs show the total energies of the signals (black line) and the contributions of the low‐ (blue line) and high‐ (red line)‐frequency components for each trial (across all subjects and channels). Subjects reported an increased level of fatigue with trials (from 1.44 ± 1.51 in the first trial to 4.22 ± 2.22 in the last trial) which are represented in the title of each graph. The center row of the figure shows three graphs with the energy of the total signal (left graph), low‐frequency component (middle graph), and high‐frequency component (right graph) for each trial after fitting each plot with a second‐order polynomial curve to facilitate comparison among fatigue levels. Although the total energy always increases during the contraction time, the energy at the beginning of the contraction increases in correlation with fatigue while the energy at the end of the contraction decreases. This effect leads to a decrease in the relative energy increment during the isometric contraction correlated to an increasing fatigue condition. This phenomenon is even more pronounced in the energy contribution of the high‐frequency component whose behavior presents larger inter‐trial variabilities. Low‐frequency component’s energy contribution, on the other hand, shows very similar inter‐trial behavior in the form of a constant linear increase. The bottom row in Figure 11 shows the evolution of the median frequency of the total signal and the low‐ and high‐frequency components for each trial. Inter‐trial statistical analysis shows no significant differences between trials’ median frequencies. However, mean values of the median frequencies for both the total signal spectrum and each spectral component presented a shift to lower frequencies correlated with the initial fatigue level.

## 4 DISCUSSION

The application of a source separation technique allowed the extraction of two components describing the spectrum of sEMG signals recorded from 12 different locations around the bicep muscle during sustained isometric contraction (Figure 9). The Bhattacharyya distance between the subspaces formed by each component and the significant difference between their median frequencies evidence high separability between them. Our results also proved that the two components can be used to describe the sEMG spectrum (720 segments per trial) with an average reconstruction rate of 84%. Spectral components’ shape and reconstruction rate agree with the results presented in Wakeling & Rozitis, (2004). The fact that similar low‐ and high‐ spectral components appear even during long‐term isometric contraction recorded on different muscle locations shows that their extraction presents robustness during fatiguing contractions and to variations in the volume conduction related to muscle location. Spectral components also present low inter‐subject variability. The 2 subjects that showed some differences (Figure 9), presented similar changes in both spectral components which suggest that the observed deviation may be related to the subject’s anatomical differences.

Figure 10 shows how the spectral decomposition allows the quantification of the signal energy shifting from high to low frequencies during the contraction period. This phenomenon, until now measured indirectly from the reduction of the signal’s median frequency, occurs similarly on all the electrodes. This further supports the idea that the spectral components represent common underlying processes not strongly influenced by recording location. In addition, our results show that high‐frequency component evolution is strongly affected by the initial fatigue level while low‐frequency component presents always similar temporal behavior (Figure 11). Even the average values of the median frequencies associated with each spectral component present a reduction correlated with increasing initial fatigue, the statistical analysis shows no significant differences. This result implies that fatigue level has little to no effect on component extraction and the shift from high to low frequencies is probably produced by changes in component modulation rather than component shifting.

The temporal behavior of the components extracted in this work can be summarized as follows. At the beginning of the contraction, subjects were asked to reach 70% of their maximum force as fast as possible. According to our results, sEMG signals produced to maintain the initial force were mainly modulated by high frequencies (Figure 10). During the contraction period there was a progressive shift in the frequency dominance, in a way that, after 30 s, the sEMG signals maintaining the contraction were modulated by significantly lower frequencies. The same overall behavior was observed regardless of the initial fatigue level, however, Figure 11 shows the effect of initial fatigue on the temporal evolution of each component. While the behavior of low frequencies was not affected by initial fatigue (progressing increment during the 30 s of sustained contraction), high frequencies presented two clear differences for increased initial fatigue. First, the energy allocated on higher frequencies at the start of the contraction was higher. Second, the decay in the high‐frequency dominance was also faster for increased initial fatigue.

Given the number of factors involved in sEMG spectral changes, trying to guess the origin of the components described in this work without any additional assessment could be highly speculative. Components extracted (Figure 9) fit the spectral distributions associated with motor unit action potentials in literature (Boonstra & Breakspear, 2012; Dobrowolski et al., 2007; Negro et al., 2015), which will support the opening statements introduced by Von Tscharner & Goepfert, (2006) and Wakeling & Rozitis, (2004). However, even if each component represented a group of spectrally separated MUAPs, with current results it is not possible to tell which underlying processes nor with which intensity was contributing to the emergence of both groups. For the moment this will remain an open question that we hope helps stimulate discussion within the scientific community.

Despite possible interpretations, a two‐component spectral decomposition proved to be a promising method for the detailed quantification of sEMG energy distribution. This methodology can be used to isolate single factors involved in sEMG spectrum modulation by quantifying their effects on high and low frequencies under proper control conditions. For example, the comparison between the current multi‐channel approach versus a single‐channel component decomposition could be used to quantify the spectral deviations produced by the recording location. Moreover, if the origins of the spectral components are clarified in the future, they will be a very useful tool for the study of physiological processes with the use of non‐invasive technologies.

## ETHICS STATEMENT

All participants received an explanation of the experimental protocol before the study and signed an informed consent agreement. This study was conducted in accordance with the Declaration of Helsinki and approved by the ethical review board of the RIKEN research institute (code of the ethical approval: Wako3 28–13).

## CONFLICT OF INTEREST

The authors state no conflict of interest.

## AUTHOR CONTRIBUTIONS

Alvaro Costa Garcia: Conceptualization; data curation; formal analysis; investigation; methodology; software; validation; visualization; writing original draft; writing review and editing. Eduardo Iáñez: Data curation; formal analysis; methodology; experiment design and writing review. Moeka Yokoyama: Conceptualization; data curation; investigation and writing review Sayako Ueda: Data curation; investigation and writing review. Shotaro Okajima: Conceptualization; data curation and writing review. Shingo Shimoda: Conceptualization; data curation; funding acquisition; investigation; methodology; writing review and editing.

## Figures and Tables

**FIGURE 12:**
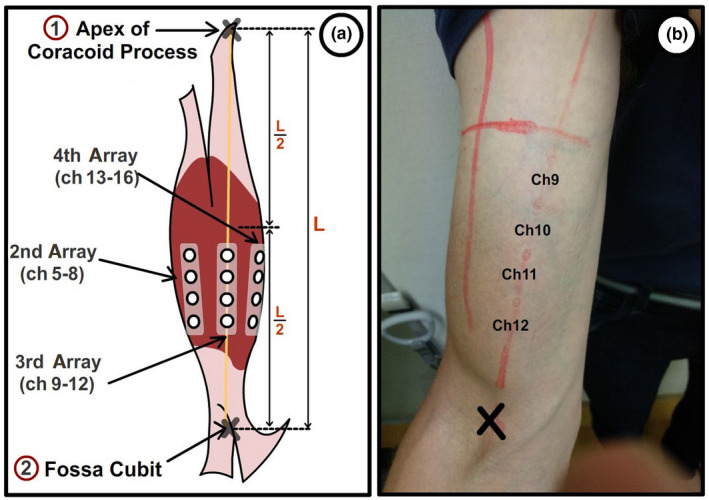
Electrode location. (a) A visual representation of the protocol followed to set the three arrays of electrodes used to measure biceps activation during isometric contraction. (b) An example of the arm of subject 7 after an experiment, with the electrode position visible from the marks left on the skin.

**FIGURE 13:**
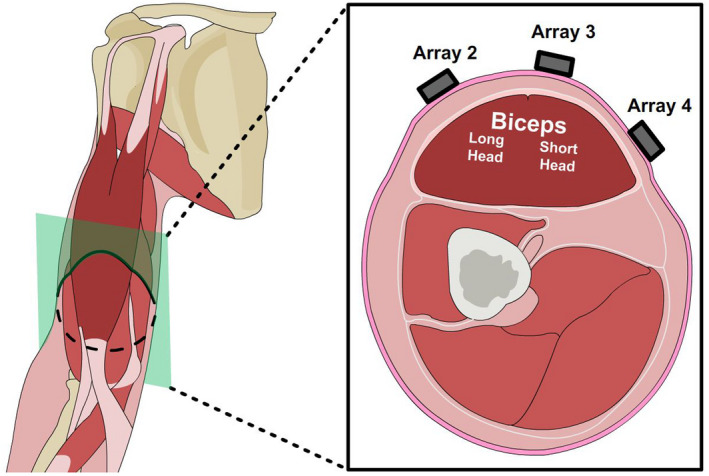
Electrode location: Cross‐section. Visual representation of a cross‐section of the arm showing the location of each array used.

## APPENDIX I PROTOCOL FOR EMG BAND PLACEMENT

Figure 12(a) shows the reference points used to select the electrode location. Point 1 corresponds to the apex of the coracoid process and Point 2 corresponds to the fossa cubit, according to the guidelines of the Surface Electromyography for the Non‐Invasive Assessment of Muscles (SENIAM) project (Stegeman & Hermens, 2007). The line connecting these two points marks the position of the short head of the biceps brachii. This line was divided into two halves to avoid simultaneous measurement on both sides of the innervated area associated with the biceps muscle (Beretta Piccoli et al., 2014). The third array of the EMG band (Channels 9–12) was aligned with the lower half of the reference line as shown in the graphical representation in Figure 12(a). Figure 12(b) shows the arm of a subject immediately after an experimental session for a realistic example of the final electrode location. The cross‐section of the arm shown in Figure 13 indicates the expected recording points of each array. As can be seen, arrays 2–4 are likely to contain more information on biceps activation.
